# Brain Metabolic PET Findings on the Long-Term Effects of COVID-19

**DOI:** 10.2967/jnumed.122.264179

**Published:** 2022-09

**Authors:** Eric Guedj, Tatiana Horowitz

**Affiliations:** * Timone Hospital, Marseille, France E-mail: eric.guedj@ap-hm.fr

**TO THE EDITOR:** We would like to thank Meyer et al. for their impressive systematic review on brain PET and SPECT findings on the acute and long-term effects of coronavirus disease 2019 (COVID-19) (1). We anticipate that this article will constitute an important reference for this topic, especially for long COVID, also by identifying roadmap points for further studies. Moreover, we believe that the authors’ recommendations are reasonable and correspond to our own practice, namely, possible use of PET/SPECT: (a) for differential diagnosis in selected cases after clinical evaluation within the framework of existing authorizations and recommendations (2), particularly for encephalitis and neurodegenerative diseases; and (b) when neurologic disorders have persisted several months, or—in the event of worsening—for cerebral assessment of such patients after a clinical evaluation to confirm such impairments, which we believe cannot be limited to the cognitive domain and consequently only to neuropsychiatric testing (e.g., dysautonomia).

We would like to clarify several points concerning our previous publications, on which recommendations for long COVID are partly based.

As accurately highlighted by our colleagues (1), the inclusion criteria of time spans from initial infection have fluctuated in our studies (3*,*^4^), albeit in accordance with the fluctuations of the French and international definitions of the condition (3 wk, 1 mo, and now 3 mo). The definition used at the time of publication was justified in our articles (3*,*^4^). We fully recognize the possible impact of this delay on PET findings and the need for further standardized studies based on the current clinical definition of long COVID. In this line, we recently showed, in a multicentric study including 143 patients, a consensual profile of brain hypometabolism on visual interpretation for approximately one half of patients with suspected neurologic long COVID approximately 11 mo after symptom onset, whereas the second half of patients had normal brain PET metabolism (5). We also agree that recommendations for the clinical use of PET imaging must take into account a delay of confirmed persistent symptoms (>3–6 mo for Meyer et al. (1)).

Meyer et al. suggested that our PET results were unjustifiably obtained with 2 distinct statistical thresholds in the 2 studies (“*P* < 0.05, FWE-corrected [familywise-error–corrected] in adults; *P* < 0.001, uncorrected in children”) (1). The same statistical thresholds were in fact used for the 2 studies (3*,*^4^). The reader can refer to the methods and Table 2 of the 2 studies (*P* [voxel]  <  0.001; *P* [cluster]  <  0.05, familywise-error–corrected) (3*,*^4^).

Meyer et al. mentioned that we reported a “weak” negative association between the number of complaints and the PET metabolism of the brain stem and cerebellum (“*r*^2^ = 0.1 and 0.34, respectively”) (1). Similarly, the reader can refer to the results of our study: the *r*^2^ was in fact 0.19 and 0.34 (*r*  =  − 0.440 and − 0.581, *P*  =  0.004 and *P* <  0.001, respectively) (3).

Meyer et al. mentioned that our hypotheses concerning metabolic modifications in long COVID changed between the 2 studies, from “neurotropism” to “inflammatory,” “dysimmune,” or “vascular” damage (1). The term *neurotropism* refers both to the direct hypothetic effects of brain viral propagation and to the possible indirect effects of the virus on inflammatory, dysimmune, or vascular damage (6). We believe that these hypotheses are well explained in our previous papers (3*,*^4^*,*^7^*,*^8^), including the one (3) quoted specifically by Meyer et al. supposedly to exclude alternative explanations (1) (immune-inflammation disorder; lesions possibly involving direct infection injury, hypoxia, and immune injuries; hypothesis of brain hypometabolic dysfunction secondary to earlier hypermetabolic inflammation; treatment of the possible inflammatory olfactive gateway and stimulation of this hypofunctional brain network). Meyer et al. also pointed out that the hypothesis of neurotropism from olfactory bulbs is independent of anosmia, since not all patients with long COVID and brain hypometabolism have functional complaints of olfactory functions. Brain impairment is not systematically associated with functional complaints, and anosognosia of olfactory deficits has been reported in patients with long COVID (9). Importantly, a recent controlled longitudinal study with MRI performed on 785 subjects before and during the outbreak demonstrated an increased reduction in gray matter thickness and tissue contrast within limbic regions connected to the olfactory regions in infected patients (6).

Meyer et al. mentioned that we considered psychologic explanation as an equal hypothesis in our last article (4) (Table 2 (1): “[s]everal possible explanations [inflammatory, immune, neurotropism, vascular, gut–brain disturbance, psychologic], but none clearly favored”). Psychologic factors were considered in our 2 previous publications as possible contributors to organic explanations and not as exclusive alternatives (possible entanglement with other factors and particularly psychologic factors (3) and possible interactions with psychologic factors (4)). Importantly, our PET results were also obtained by comparing long-COVID patients with age-matched control patients with functional symptoms, in whom somatic cerebral diseases were thereafter excluded at follow-up (4), bringing additional arguments against exclusive psychologic explanations. We also noticed that this profile is distinct from those associated with the lockdown impact (10).

Finally, Meyer et al. proposed an interesting methodologic discussion on various postprocessing choices, including the tricky issue of activity normalization, with the proposal to further develop principal-component analyses. Such considerations have been extensively discussed regarding possible advantages and limits, as have discrepancies among studies in addition to the heterogeneity of patients (7).
